# Experiences and Perspectives of Individuals with Cystic Fibrosis and Their Families Related to Food Insecurity

**DOI:** 10.3390/nu14132573

**Published:** 2022-06-21

**Authors:** Montserrat A. Corbera-Hincapie, Samar E. Atteih, Olivia M. Stransky, Daniel J. Weiner, Iris M. Yann, Traci M. Kazmerski

**Affiliations:** 1 Department of Pediatrics, University of Pittsburgh School of Medicine, Pittsburgh, PA 15213, USA; atteihse@upmc.edu (S.E.A.); daniel.weiner@chp.edu (D.J.W.); iris.yann@chp.edu (I.M.Y.); traci.kazmerski@chp.edu (T.M.K.); 2 Center for Innovative Research on Gender Health Equity (CONVERGE), Pittsburgh, PA 15224, USA; ols25@pitt.edu

**Keywords:** cystic fibrosis, food insecurity, experiences

## Abstract

Food insecurity (FI) rates among people with cystic fibrosis (CF) are significantly higher than in the general US population. This study explored the experiences and perceptions of adults and parents of children with CF surrounding FI. We recruited parents of children with CF ages 0–18 years and adults with CF ages 18 years and older from a large, accredited U.S. CF care center and the Cystic Fibrosis Foundation Community Voice to participate in a qualitative study using semi-structured telephone interviews to explore experiences and preferences related to food insecurity. Two coders independently reviewed each transcript to apply the codebook and identify any emerging codes using an ongoing, iterative process to identify central themes. We interviewed 20 participants (six adults with CF and 14 parents of children with CF) and identified five major themes: (1) FI in CF is influenced by a variety of factors, ranging from nutritional demands to competing financial barriers; (2) FI impacts CF health outcomes; (3) Open patient-provider communication around FI is vital; (4) FI screening and discussions should be normalized in CF care; (5) Comprehensive FI resources are vital. FI is an important topic that should routinely be addressed with the CF care team to destigmatize and encourage individuals to be more forthcoming about their FI status. Results from this study will inform future larger investigations on the impact of FI on CF health and aid in the design and planning of targeted interventions and advocacy efforts.

## 1. Introduction

Food insecurity (FI), defined as a household condition in which access to adequate food is limited by lack of money and other resources, is an important health concern that has the potential to impact on children and families in a dramatic way [1]. In 2020, 10.5 percent of households in the United States (US) were food insecure, and nearly 15 percent of households with children were food insecure [2]. FI, especially in households with children, has since been exacerbated by the COVID-19 pandemic [3]. Furthermore, individuals living with chronic illnesses in the US have an increased prevalence of FI [4].

FI rates among people with cystic fibrosis (CF) are significantly higher than the general US population [5]. Ideal nutritional status, defined as body mass index (BMI) at or above the 50th percentile for children and adolescents aged 2 to 20 years or BMI of at least 22 and 23 for females and males respectively, has been found to correlate with improved long-term linear growth, pulmonary function, and long-term survival in CF. As such, the CF Foundation (CFF) recommends an intake of at least 500 calories per day over the standard daily requirement with fat intake composing about 35–40% of those calories [6,7,8]. Given that people with CF have increased caloric demands, FI may contribute to the inability to achieve and maintain appropriate weight gain and lead to malnutrition in CF and is a nutritional risk factor for the CF population [9,10,11,12].

The individual and family experience of FI in CF, where diet and nutrition are so heavily valued and monitored, has not been previously studied. We explore the lived experiences of both adults with CF and the parents of children with CF, who have reported experiencing FI on routine screening. We investigated perceptions surrounding FI screening, patient-provider interactions, and emotions surrounding disclosure with the overarching goal of improving identification and provision of resources to those experiencing FI in CF.

## 2. Materials and Methods

### 2.1. Study Participants

We recruited parents or primary caregivers of children with CF ages 0–18 years and adults with CF ages 18 years and older using two methods. First, we recruited participants from a large, accredited U. S. CF care center during outpatient visits between November 2020 and May 2021 who reported experiencing FI on verbal screening using a validated screening tool. We also recruited participants who self-reported FI in June 2021 using a national convenience sample from the CFF Community Voice (CV), a virtual avenue for people with CF and their family members to share their experiences and perspectives on CF research and care [13]. We used pseudonyms to represent study participants.

### 2.2. Interviews and Data Collection

The principal investigator (MACH), a pediatric gastroenterologist and health services researcher, conducted the interviews in a private space using a secure telephone line. The interviewer was not directly involved in the participants’ clinical care. We collected participant demographic information, including age, race, and gender of the person(s) with CF and structured interviews by key open-ended questions. The interviews explored the participants’ experiences surrounding FI, their understanding of the impact of FI on their or their child’s health, their experiences discussing FI with the CF team and other healthcare providers, their coping strategies for addressing FI, and their perspectives on what resources were needed to better support those with CF facing FI (Box 1).

The institutional review board (IRB protocol #20080145) approved the study. Patient consent was waived due to the study’s anonymous low-risk nature. We compensated each participant with a $50 gift card. All interviews were conducted in English and audio recorded. Interviews lasted between 25 and 60 min. We reached thematic saturation after the 10th interview, suggesting that our total sample was adequate for establishing important themes as they relate to FI in CF.

A co-investigator (TMK), a CF physician and health services researcher with extensive experience on qualitative research, served as a resource during the entire data collection process.

Box 1 **Key Interview Questions.**

- Can you tell me more about your experiences with food insecurity?
- In the past 12 months, have you worried about paying the rent/mortgage, falling behind on utilities, facing transportation or health care barriers? Tell me more about this.
- How do you think food insecurity has affected your [or your child’s] health?
- Did you ever bring up food insecurity with any health care provider? Tell me more about this.
- Should the CF team be asking about food insecurity? Why or why not?
- Has anyone on your CF care team (i.e., your doctor, nutritionist, nurse, social worker, etc.) ever talked to you about food insecurity? How did this conversation go?
- What resources would be helpful for you?
- Have you ever been offered resources during an office clinic? If so, which did you find the most helpful and why?
- How can your CF team better support people with CF with food insecurity?
- What advice do you have for other parents with children with CF or individuals with CF, who are experiencing food insecurity?
- What advice do you have for your CF team on how to make these discussions more comfortable?
- What coping strategies do you have related to food insecurity?
- What is the best way to screen for food insecurity? Tell me more about this.
- If you have questions about food insecurity that are specific to CF, where do you go for information?
- In the future, how would you want to hear about any information regarding food insecurity?

CF: cystic fibrosis.

### 2.3. Data Analysis

We transcribed the interviews and used Dedoose software to facilitate data management and coding. The primary investigator (MACH) developed an initial codebook based on the interview guide. MACH and a second coder (SA), a pediatric pulmonologist, then independently reviewed each transcript to apply the initial codebook and identify any emerging codes. The coders reviewed their coding together and defined any new codes after the first few transcripts were independently coded. This approach was used in an ongoing, iterative process. The coders applied the final codes to all transcripts, and the study team identified central themes and representative quotations [14,15,16].

## 3. Results

We interviewed 20 participants (six adults with CF and 14 parents of children with CF). We recruited 15 participants directly from clinic and five from CV. Table 1 includes participant demographic information. Major themes and subthemes with representative quotations are below.

### 3.1. Theme 1: FI in CF Is Influenced by A Variety of Factors, Ranging from the Nutritional Demands to Competing Financial Barriers

Many participants expressed significant concerns surrounding the expense of a CF diet, due to increased caloric requirements and increased need for expensive food items, such as proteins and fats. Half of the participants felt that they purchased lower quality food due to these considerations. “Avery”, a parent of three boys with CF, discussed their family’s monthly food budget and stated, “It’s a tricky thing because I know they need to eat more, a lot more, but how much money can you put into a grocery budget…things that they need that are gonna be good for their diet are also the expensive things”.

Most participants felt that there were several competing barriers that compounded the effects of FI, including basic living expenses (i.e., utilities, rent), single income households, healthcare costs, transportation barriers, and households with multiple children with CF. Participants expressed the difficulty of having to choose between food and competing costs. “Riley”, a parent of two children with CF, shared “I don’t make a whole lot of money, but I also don’t qualify for food stamps. Everything that I buy I pay for myself. It gets hard on a single [parent]”. “Morgan”, age 38, shared their experiences with transportation costs and stated, “I do pulmonary rehabilitation at the hospital three times a week, so driving back and forth, that takes gas and a lot of things. I usually end up just barely making it, month to month”. “Avery” shared that having three children with CF is “going to be like I’m feeding six boys…they need twice as much food as the average kid”.

Most participants felt that COVID-19 was an external contributor to FI. “Emerson”, a parent of two girls with CF, shared that due to COVID-19, their family “ended up having to pick between if we were paying bills or buying groceries because we were already rammed with credit card debt”. The pandemic worsened FI for some families and led to FI in some families that had not experienced it previously.

### 3.2. Theme 2: FI Impacts CF Health Outcomes

Nearly all participants felt that FI played a role in their own or their children’s weight with an emphasis on being underweight. “Avery” reflected on how FI has impacted their children’s weight by stating “we do try to maintain their health as best within our power, but I think they could probably do so much better with their weight if I had the means to give them what I wish I could as far as food”.

Some participants felt that FI also played a role in their lung function and susceptibility to infections. “Dallas”, age 37, reflected on how they “had more [lung] infections…getting sicker a lot more” when they were underweight due to FI. “Avery” said “If they could just have what they need to do better, and just nutrition …It’s just everything rides on that ’cause they’ll have less hospitalizations if they’re nutritionally better. I just hope that this is somethin’ that gets more talked about, and more taught to the professionals”.

### 3.3. Theme 3: Open Patient-Provider Communication around FI Is Vital

Most participants felt that FI was associated with embarrassment and guilt. This emotional impact led to difficulty and fear of disclosing FI status and asking for help. “Riley” noted that “it does get embarrassing being asked … it’s hard to say yes…that you’re struggling…sometimes it’s just hard for people to say that they are”. “Charlie”, a parent of two girls with CF, said that “there’s a feeling like you’re failing, or some desperation” when disclosing FI and accepting resources.

Almost half of the participants expressed difficulty in disclosing FI because they felt undeserving of help and that other families or patients could use FI resources more. “Avery” shared, “I don’t want to put ‘yes’ on [the FI screener] knowing there’s probably people who can’t even get their kid one package of bacon. I feel like those resources should go to those people, but I don’t know what you do about the people in the middle who don’t qualify for SNAP [Supplemental Nutrition Assistance Program] but aren’t quite able to get what they need”.

Most participants felt strongly that overcoming this fear of asking for help and accepting FI resources were the best ways to advocate for loved ones or oneself. “Brooklyn”, parent of a boy with CF, expressed, “[Don’t be] scared to say something because [the CF providers] don’t know what’s going on unless you bring it to their attention. Don’t be scared to speak up because this is your child’s voice right now”.

### 3.4. Theme 4: FI Screening and Discussions Should Be Normalized in CF Care

Many participants felt that normalizing FI and standardizing how the topic is approached and discussed would encourage people to be more forthcoming about their FI status. “Cameron”, a parent of a toddler with CF, shared that it would be helpful to have a universal FI screener on registration that is “worded nicely... so that it would just be like, this is what we do. It’s part of the process. We are not singling anyone out”.

Almost every participant felt that the CF team should be screening for FI at every visit. “Dallas” stated that “there are a lot more people that are struggling [with FI] in the CF community than they [CF care team] know of”. The majority also felt that nonverbal (i.e., questions on a handout or on a tablet) or a combination of nonverbal and verbal screening is preferred over verbal screening (i.e., staff asking on arrival to visit) alone. “Hayden”, parent of a boy with CF, expressed that a nonverbal method of screening would be ideal because “some people are embarrassed about what they make …and some people might not be open” if asked verbally.

Participants noted that FI screening can miss those who do not qualify for governmental assistance or whose income is irreflective of FI status. “Jesse”, a parent of a girl with CF, expressed “[We were] never eligible to receive assistance because of [my] husband’s salary. It’s like even though he made a certain amount, it still didn’t reflect how [we] were struggling”.

The majority of participants felt that FI discussions should be with either the dietitian or social worker. “Remy”, parent of a transgender male with CF, said “I feel comfortable with the social worker. They’re there to help you and make sure everything’s okay and they’re less intimidating I think than maybe a doctor or nurse”. “Sam”, age 34, felt that “the dietitian should always be the one to initiate the conversation because they’re the ones trained to understand the capacity of food, what food deserts are, what FI does from a nutritional standpoint”.

Furthermore, about a third of participants felt that CF physicians have limited understanding of FI or should not be burdened with such non-medical aspects of care. “Riley” stated “[Physicians] should be more concerned about the problems that are going on, the things that can be done to help health-wise”. “Emerson” also shared “it’s hard to discuss FI … and it’s a difficult topic to get too personal with doctors, and frankly, I don’t think they really understand either”.

### 3.5. Theme 5: Comprehensive FI Resources Are Vital

The majority of participants felt that food banks, SNAP, and gift cards were helpful resources. “Hayden” stated, “The gift cards definitely helped because I can get gas, and that saves money for other things. We receive some food stamps…that definitely helped during the pandemic”. Most preferred that resources be provided via an electronic method (i.e., email) as opposed to paper. “Riley” explained how “electronically is easier because papers get lost. Emails you can always find”.

Most participants felt strongly that family served as a great support network. “Morgan” expressed “I have a great support system. I think that’s part of the reason why I’m alive. I have a family that has taught me to fight. I have a family that loves me and my significant other loves me very, very much”.

## 4. Discussion

This is the first study to explore the perspectives and experiences of people with CF and families with children with CF related to FI. Participants expressed that FI is influenced by a variety of factors, ranging from the increased CF nutritional demands to competing financial barriers, and can lead to poor CF health outcomes. FI also elicited feelings of embarrassment and guilt that led to hesitancy in disclosing FI status. Given this, most participants felt that this was an important topic that should routinely be addressed with the CF care team to destigmatize and encourage individuals to be more forthcoming about their FI status so that they can be connected to the appropriate resources.

Our results highlight important barriers to optimal adherence to CF dietary recommendations that can be exacerbated by FI, including cost of food, increased caloric requirements, single income household, transportation, and cost of housing, utilities, and healthcare. A recent study by our team showed that people with CF living in and near food deserts, a risk factor for FI, had increased odds of with a non-ideal lower BMI or weight-for-length [12]. It has also been noted that FI contributes to greater healthcare costs and financial strain, particularly when looking at patients with chronic disease [17]. Food-insecure individuals with inflammatory bowel disease had higher “financial toxicity”, defined as “financial hardship due to medical bills, personal and health-related financial distress, cost-related medication nonadherence, and healthcare affordability [18]”.

FI is intricately associated with socioeconomic status (SES), and studies have shown an increased risk of mortality from CF before the age of 18 years in people with lower SES [19,20,21]. People with CF, particularly those with FI, report high rates of cost-related medication underuse, delayed health care visits, and eating less [22]. Additionally, a recent study linked respiratory health in people with CF to state- and area-level characteristics, particularly an association with area resource deprivation and overall state child health [23]. With these prior studies in mind, our results help us understand further why individuals with CF might have difficulty in adhering to dietary recommendations, which can lead to poor health overall. While SES screening can be challenging, universal FI screening may uncover those struggling to adhere to CF dietary recommendations.

Another important factor that most participants felt contributed significantly to their FI was the COVID-19 pandemic, which has led to unprecedented levels of FI due to increased food prices, disrupted community support networks, increased rates of unemployment and school/daycare closures [24,25]. The pandemic has also led to increased reports of worse mental health and self-care in food-insecure persons with CF [26]. The CFF recently conducted a national survey to assess trends in FI screening and interventions prior to and during the pandemic. FI screening increased during the pandemic and demonstrated an overall increase in FI rates, exceeding the national average in some centers. Some pediatric programs reported that 20% to 43% of their patients experienced FI during the pandemic [27].

We found that embarrassment about FI status often led to decreased disclosure to CF teams. The role of stigma in food inequities is understudied and is manifested at the individual and structural level [28]. A recent study examining the relationship between FI and adults’ experiences of neighborhood safety and discrimination during the COVID-19 pandemic showed that food-insecure adults reported higher rates of interpersonal racism and disrespect compared to food-secure adults [29].

Patient-provider communication is key when discussing FI, and provider education is paramount. Physicians are trained to manage acute and chronic illness but are often less adept at screening and addressing social determinants of health (SDH), creating a significant gap in comprehensive care. As SDH, such as FI, contribute to health outcomes, medical training should incorporate SDH to improve provider comfort and competence in addressing FI thoroughly and sensitively. By building appreciation for the underlying social structures that contribute to poor health outcomes, such training can improve providers’ cultural competency [30]. Recognizing the need for good patient-centered communication on this topic, the CFF recently published a discussion guide for CF providers related to FI.

This study did have limitations. We interviewed a small sample of participants (although thematic saturation was reached with our sample size of 20), which may limit generalizability. Additionally, one-quarter of our sample was recruited from CV, leading to self-selection bias with increased engagement, as these participants are active in CF advocacy and research. Additionally, there was unintentional lack of heterogeneity in participants’ race/ethnicity, as most participants were White, resulting in a limited perspective from minority groups. Given that most of our participants were recruited from one center, there was little geographic diversity that might account for differences in how CF care centers approach FI screening and discussions; however, 25 percent of study participants were recruited through CV, which does include geographically diverse participants and can account for possible differences in how FI is approached by different CF care teams and regions. The positionality of the interviewer, particularly employment (i.e., physician), may have influenced the interview interactions, as participants may have been embarrassed sharing FI experiences; however, participants were aware that the interviewer was a physician prior to enrolling in the study, and many expressed gratitude that a physician was involved in a study exploring FI.

## 5. Conclusions

Results from this study will inform future larger investigations on the impact of FI on CF health and aide in the design and planning of targeted interventions and advocacy efforts. Findings may also be applicable to other chronic diseases that are impacted by nutrition. By gaining the perspectives of these individuals, we can develop patient-, provider- and systems-based interventions, including educational resources, provider training, and standardized screening practices.

## Figures and Tables

**Table 1:** Characteristics of the CF patient sample.

Participant Characteristics	Mean (SD) or % (*n*)
**People with CF***n* = 6	
Age	32 (range 18–38)
Gender	
Female Male	67% (4) 33% (2)
Modulator use	
Yes No	67% (4) 33% (2)
Race/Ethnicity	
White Black Multiracial (White and Native American)	67% (4) 17% (1) 16% (1)
**Parents***n* = 14	
Age of children with CF *	10.3 (range 2–19)
Gender of children with CF	
Female Male Transmasculine	43% (9) 52% (11) 5% (1)
Gender of parents participating	
Female	100% (14)
Modulator use in children	
Yes No	52% (11) 48% (10)
Race/Ethnicity	
White Black	95% (20) 5% (1)
Households with multiple children with CF	43% (6)

*, 21 children total.

## Data Availability

The data presented in this study are available on request from the corresponding author.
